# Cardiac resynchronization considerations in left bundle branch block

**DOI:** 10.3389/fphys.2022.962042

**Published:** 2022-09-15

**Authors:** Nathan W. Kong, Gaurav A. Upadhyay

**Affiliations:** ^1^ Department of Internal Medicine, University of Chicago Medicine, Chicago, IL, United States; ^2^ Section of Cardiology, Center for Arrhythmia Care, University of Chicago Medicine, Chicago, IL, United States

**Keywords:** left bundle branch block, cardiac resynchronization therapy, biventricular pacing, conduction system pacing, left bundle branch area pacing

## Abstract

Cardiac resynchronization therapy (CRT) via biventricular pacing (BiVP) is an established treatment for patients with left ventricular systolic heart failure and intraventricular conduction delay resulting in wide QRS. Seminal trials demonstrating mortality benefit from CRT were conducted in patients with wide left bundle branch block (LBBB) pattern on electrocardiogram (ECG) and evidence of clinical heart failure. The presence of conduction block was assumed to correlate with commonly applied criteria for LBBB. More recent data has challenged this assertion, revealing that LBBB pattern may include distinct underlying pathophysiology, including patients with complete conduction block, either at the left-sided His fibers or the proximal left bundle, intact Purkinje activation with wide LBBB-like QRS, and patients demonstrating both proximal block and distal delay. Currently, BiVP-CRT is indicated for all QRS duration ≥150 ms and may be considered for BBB patterns from 130 to 149 ms with robust clinical data to support its use. Despite this, however, there remains a significant number of non-responders to BVP. Conduction system pacing (CSP) has emerged as an alternative approach to deliver CRT and correct QRS in patients with conduction block. Newer hybrid approaches which combine CSP and traditional BiVP-CRT and may hold promise for patients with IP or mixed-level block. As various approaches to CRT continue to be studied, physiologic phenotyping of the LBBB pattern remains an important consideration.

## Introduction

For over a century, left bundle branch block (LBBB) has been recognized as a clinical entity. The clinical significance of LBBB has changed over the years from being first perceived as a non-harmful electrocardiogram (ECG) finding to more recently being associated with poorer prognosis, particularly in patients with severe, symptomatic heart failure (Master et al., 1940; Tabrizi et al., 2007). Cardiac resynchronization therapy (CRT) via biventricular pacing (BiVP) has been established to reduce mortality in patients with electrical dyssynchrony and left ventricular systolic dysfunction (Moss et al., 2009; Zareba et al., 2011; Gold et al., 2012; Goldenberg et al., 2014). Large clinical trials have shown that the presence of LBBB on surface ECG is one of the best predictors of CRT response (Linde et al., 2008; Moss et al., 2009). Multiple guidelines have adopted this definition and emphasized presence of LBBB as a criterion when selecting patients for CRT (Tracy et al., 2012; Glikson et al., 2021; Allaw et al., 2022). However, despite this, there remains a significant percentage of patients who do not experience reduction in morbidity or mortality from CRT (Mullens et al., 2009). Recent observations of suggest that the LBBB pattern recognized on surface ECG may distinct underlying pathophysiology: complete conduction block through the left bundle of the His-Purkinje system, wide LBBB-like QRS due to left ventricular hypertrophy or diffuse fibrosis leading to interventricular conduction delay with preserved His-Purkinje activation (IVCD with IPA), or a combination of both proximal block and concomitant distal disease (Upadhyay et al., 2019a; Tung and Upadhyay, 2020). CRT response has been shown to depend on the mechanism of the bundle branch block (Caputo et al., 2018; Jastrzębski et al., 2018). Conduction system pacing (CSP) and hybrid pacing are emerging strategies for CRT but may not be suitable for all types of LBBB patterns.

## How to define left bundle branch block?

### Surface ECG

Most definitions of LBBB are based on surface ECG patterns. The American Heart Association (AHA), American College of Cardiology (ACC), and Heart Rhythm Society (HRS) collaborated on the currently adopted definition in 2009 and was restated in 2018 (Surawicz et al., 2009; Kusumoto et al., 2019). By their definition, the LBBB pattern is defined by a QRS duration ≥120 ms with a broad notched or slurred R-wave in leads I, aVL, V5, and V6; delayed time to R-wave peak; absent Q waves; ST segment and T-wave changes. Importantly, the precise definition of some of the components of this definition, including the assessing timing of slurs or notches, remains subjective.

In 2011, the landmark Multicenter Automatic Defibrillator Implantation with Cardiac Resynchronization Therapy (MADIT-CRT) trial suggested that patients with reduced ejection fraction <30% and prolonged QRS >130 ms derived significant benefit in composite heart failure events and death from CRT-D compared to ICD if they had a LBBB pattern compared to non-LBBB (i.e.,: right bundle branch block or nonspecific interventricular conduction delay) (Goldenberg et al., 2014). Notably, the study was underpowered to assess death alone as there was a trend but no significance in subgroup analysis. The MADIT-CRT study altered the definition of LBBB pattern and incorporated the presence of a QS or rS pattern in lead V1 and removed the criteria of delayed R-wave peak and ST segment and T-wave changes (Zareba et al., 2011). This definition was adopted by the European Society of Cardiology (ESC) in 2013 and incorporated into their guidelines (Glikson et al., 2021; Brignole et al., 2013).

Strauss and colleagues in 2011 observed a growing body of evidence that CRT had greatest benefit in patients with LBBB due to conduction block rather than hypertrophy, underscoring the importance of accurate patient identification. By reviewing serial ECGs and with the understanding that hypertrophy was a gradual process whereas conduction block was an abrupt process, the authors proposed a modification to the LBBB criteria. What later was known as the Strauss criteria, they suggested that LBBB be more stringently defined as a QRS duration of ≥130 ms in women and ≥140 ms in men; a QS or rS pattern in lead V1 and V2; and *mid*-QRS notching or slurring in at least two of the leads V1, V2, V5, V6, I, and aVL (Strauss et al., 2011).

When compared systematically in a large series of patients, however, no single set of criteria was superior at identifying response to CRT (van Stipdonk et al., 2020). Given the absence of a “gold standard” for determining whether a conduction block is present or not, there may be inherent limitations to the use of surface ECG in predicting patient response to CRT (Perrin et al., 2012; Caputo et al., 2018; Jastrzębski et al., 2018). Indeed, up to one third of the patients who meet ACC/AHA/HRS criteria for LBBB demonstrated intact activation of their ventricles via the His-Purkinje system (Auricchio et al., 2004).

### Intracardiac electroanatomic mapping

While distinguishing LBBB due to conduction block versus IVCD with IPA may be challenging on surface ECG, intracardiac recordings of ventricular activation patterns can more readily distinguish these two entities. In 1984, Vassallo et al., used endocardial catheter mapping on patients with LBBB on surface ECG and concluded that left ventricular endocardial activation was heterogenous (Vassallo et al., 1984). Several decades later in 2003, Rodriguez et al., used 3D endocardial mapping systems and observed 2 types of septal activation patterns in patients with LBBB and heart failure (Rodriguez et al., 2003). Auricchio and colleagues utilized non-contact mapping to identify patterns of ventricular activation in patients with LBBB pattern, and similarly concluded that transseptal conduction was normal in one-third (Auricchio et al., 2004). Subsequently, Derval et al., used high density electroanatomic mapping to demonstrate that patients with LBBB had distinct left ventricular activation patterns compared to patients with nonspecific intraventricular conduction delay (Derval et al., 2017). More recently, Upadhyay and colleagues employed multielectrode mapping catheters to assess left-sided septal activation among 72 patients with LBBB. They found that the mechanism of LBBB pattern was due to complete conduction block (CCB) within the proximal left conduction system in 64% of patients, while intact Purkinje activation (IPA)—a finding usually attributed to IVCD—was noted in the remaining 36%. Importantly, the population studied included patients both referred for conventional device indications along with those referred for electrophysiology (EP) study in the treatment of ventricular tachycardia, and therefore may have included patients more advanced disease (Upadhyay et al., 2019a). Regardless, the conclusion from these studies is that determining conduction block from surface ECG alone remains limited.

While assessing LBBB via intracardiac mapping remains the most rigorous modality to assess for CCB, it is generally only utilized in patients who already have another indication for EP study. It is unclear whether routinely performing left-sided septal mapping as an isolated diagnostic study in the absence of concomitant indications is justified due to the incremental risks of arterial access. The risk profile of left ventricular septal mapping is likely comparable to routine diagnostic coronary angiography with left ventricular pressure assessment. An additional barrier may be the justification of economic costs and time associated with EP study to guide CRT implant. Presently, there are no published guidelines to suggest which patient populations would most benefit from invasive studies to further clarify their electrical activation pattern.

### Transthoracic echocardiogram

One of the main hypotheses as to why CRT improves mortality in patients with heart failure is because LBBB causes left ventricular (LV) dyssynchrony thus leading to unfavorable cardiac remodeling. Thus, transthoracic echocardiogram (TTE) has been used as a non-invasive tool to visually assess for LV dyssynchrony which are correlated with conduction block from complete LBBB. There are a number of TTE patterns which are indicative of this degree of LV dyssynchrony including septal flash or septal beak (the interventricular septum [IVS] rapidly and briefly moves leftward before or during ejection); septal systolic rebound stretch (IVS elongates during early systole); apical rocking (apex moves towards septum during early contraction and then swings to the lateral wall during late contraction); LV lateral wall hypertrophy with late contraction; or short filling and ejection times with a longer isovolumic phase due to late aortic valve opening (DILLON et al., 1974; Voigt, 2015; Walmsley et al., 2016). Contraction pattern assessment by 2-dimensional strain echocardiography to assess for typical LBBB mechanical activation has been shown to improve selection of CRT responders when used in conjunction with surface ECG (Risum et al., 2015). Tissue doppler index and 3D approaches have also been studied to assess for LV dyssynchrony (Dohi et al., 2005; Marsan et al., 2008; Van de Veire et al., 2008; Surkova et al., 2017). It has been shown that TTE patterns of LV dyssynchrony may play a role in identifying CRT responders, and correction of these patterns after CRT is independently associated with lower all-cause mortality and reverse LV remodeling (Stankovic et al., 2016). In fact, two randomized trials showed that echo guided placement of the LV lead to areas of imaged dyssynchrony improved rates of death and HF hospitalization (Khan et al., 2012; Saba et al., 2013).

There are several ‘mimics’ to LBBB induced LV dyssynchrony on TTE, particularly for septal flash. These ‘mimics’ include coronary artery disease, post-cardiac surgery, right ventricular dysfunction, severe mitral stenosis, pericardial disease, other conduction system abnormalities (e.g., ventricular pacing, pre-excitation, premature contractions), or extra-cardiac posterior compression (e.g., hiatal hernia, pregnancy, ascites) (Surkova et al., 2017). Additionally, the presence of these patterns may be difficult to elucidate, particularly in cases of borderline dyssynchrony. Finally, while TTE patterns of dyssynchrony may be valuable visual representations of underlying physiology, there has been no study to day showing that TTE patterns of dyssynchrony predict response to CRT. In fact, the presence of LV dyssynchrony alone, in the absence of wide QRS, does not predict CRT response. This was shown in the 2013 EchoCRT trial which randomized 809 patients with QRS < 130 ms and TTE evidence of LV dyssynchrony (Ruschitzka et al., 2013). The trial was terminated early due to increased mortality observed in the patients randomized to CRT, and this observation of harm from BiVP in narrow QRS patients is perhaps one of the most important findings reported among CRT trials in the last decade.

### Emerging modalities

Given the interest in accurately defining LBBB to identify patients who would benefit from CRT, there are several emerging modalities presently being explored. The use of ultra-high frequency ECG (UHF-ECG) has shown early promise in increasing accuracy of patient selection for CRT. UHF-ECG utilizes much higher acquisition frequencies to allow for a much more detailed understanding of electrical activity during ventricular depolarization (corresponding to the QRS complex seen on standard 12-lead ECG, along with additional recording at sites V7, V8, and V9) (Jurak et al., 2017; Jurak et al., 2020). The main limitations of UHF-ECG include the requirement of new hardware and lack of validation against intracardiac assessment of LBBB.

Another area of active interest is the use of non-invasive body surface mapping to improve accuracy in identifying ventricular dyssynchrony beyond QRS duration and morphology. Ploux and collegues studied the use of non-invasive mapping in 2013 and found ventricular electrical uncoupling on body mapping predicted clinical CRT response better than presence of LBBB on standard surface ECG (Ploux et al., 2013). Similarly, other groups are studying machine learning algorithms that may be harnessed to identify surface ECG waveforms beyond LBBB morphology and QRS duration which may better predict CRT response (Feeny et al., 2020). Recently, QRS area has been shown to be associated with clinical and echocardiographic response to CRT (van Stipdonk et al., 2018; Emerek et al., 2019). Okafor et al. even found that QRS area was superior to QRS duration and QRS morphology in predicting mortality after CRT (Okafor et al., 2019).

Advanced cardiac imaging technique have also been explored as non-invasive ways to better characterize LV dyssynchrony. Single photon emission computed tomography (SPECT) and 18F-fluorodeoxyglucose positron emission tomography (PET) studies have demonstrated that LBBB results in relative reduction in coronary flow to the septum and hyperperfusion to the lateral wall (Koepfli et al., 2009; Claridge et al., 2015). Other techniques such as quantitative gated SPECT have shown to feasibly demonstrate LV dyssynchrony and predict CRT response with a sensitivity of 83% and a specificity of 81% (Boogers et al., 2009). Similar to TTE, cardiac magnetic resonance imaging (CMR) can be utilized to assess for mechanical LV dyssynchrony using strain patterns, timining of myocardial thickness, and volume changes. CMR has superior imaging resolution as compared to TTE and can additionally provide information on the extent of myocardial scar and detailed anatomy of the coronary veins, both important in the planning of CRT (Sohal et al., 2014; Surkova et al., 2017). The major advantages and limitations of each modality is outlined in Table 1.

**TABLE 1:** Strength and limitations of different modalities to characterize left bundle branch block (LBBB) pattern.

	Strengths	Limitations
Surface electrocardiogram (ECG)	- Easy to complete	- Unable to differentiate between different types of conduction block patterns (ie: CCB vs. IVCD with IPA)
	- Inexpensive	- Multiple different definitions from different societies and studies
	- Non-invasive	- The interpretation of “notching” or “slurring” used in the definition can be subjective
	- Does not require extensive expertise to interpret	
	- Large high quality randomized control trials have used surface ECG criteria	
	- Multi-societal, international guidelines have standardized definitions	
	- Most robustly studied	
Intracardiac electroanatomic mapping	- Can distinguish different types of LBBB patterns	- Invasive and expensive
	- May improve selection of patients who would benefit from CRT	- Time intensive
	- May improve selection of patients who would benefit from CSP or hybrid pacing strategies	- No study to demonstrate improvement in patient selection for CRT
		- Incremental risks relative to device-only implant
Transthoracic echocardiogram	- Non-invasive	- Requires specialized training for interpretation
	- Routinely completed as part of workup for heart failure	- No highly established guidelines for definitive LV dyssynchrony
	- Able to visualize mechanical LV dyssynchrony which may enhance selection of patients who would benefit from CRT	- CRT for the presence of LV dyssynchrony in the absence of wide QRS duration has been shown to increase mortality—thus questioning the relative specificity of measures
Ultra-high frequency electrocardiogram	- Non-invasive	- Requires new hardware and specialized training
	- Would be an extension of existing modalities (ie: surface ECG)	- Cannot specifically distinguish septal activation pattern
	- May improve patient selection for CRT given higher level of detail of ventricular depolarization	- Lack of large validation studies
Surface body mapping	- Non-invasive	- Requires new hardware and specialized training
	- May improve patient selection for CRT response	- Higher costs than current approaches, and often requires concomitant CT imaging
		- Primarily reflects epicardial activation and cannot specifically distinguish septal activation pattern
		- Lack of large validation studies
Cardiac computed tomography	- Noninvasive	- Requires specialized equipment and hardware
	- Patterns of mechanical LV dyssynchrony may enhance selection of patients who would benefit from CRT	- Higher costs
	- Allows for high resolution evaluation of coronary sinus venous anatomy	- Requires contrast and may be limited in patients with chronic kidney disease
		- Requires specialized training for interpretation
		- Lack of established criteria to predict CRT response
Cardiac magnetic resonance imaging	- Noninvasive	- Requires specialized equipment and hardware
	- Higher resolution of mechanical LV dyssynchrony patterns as compared to TTE	- Likely more costly
	- Can give greater resolution of anatomic considerations and burden of myocardial scarring	- Requires specialized training for interpretation
	- Often obtained in the workup of heart failure and provides insight into mechanism of underlying myopathy—dense septal scar may be used to prognosticate on success from CSP	- Limited image acquisition in patients with pre-existing devices requiring upgrade
Artificial intelligence	- Can run in the background of current clinical care	- Unknown costs
	- May be able to identify patterns beyond LBBB morphology and QRS duration to improve patient selection for CRT	- Requires new software and data acquisition protocols
		- Requires large datasets to establish measures which have yet to be obtained

CCB, complete conduction block; CT, computed tomography; IPA, intact Purkinje activation; IVCD, intraventricular conduction delay; CRT, cardiac resynchronization therapy; CSP, conduction system pacing; LV, left ventricle.

## Anatomic considerations of LBBB

**FIGURE 1:**
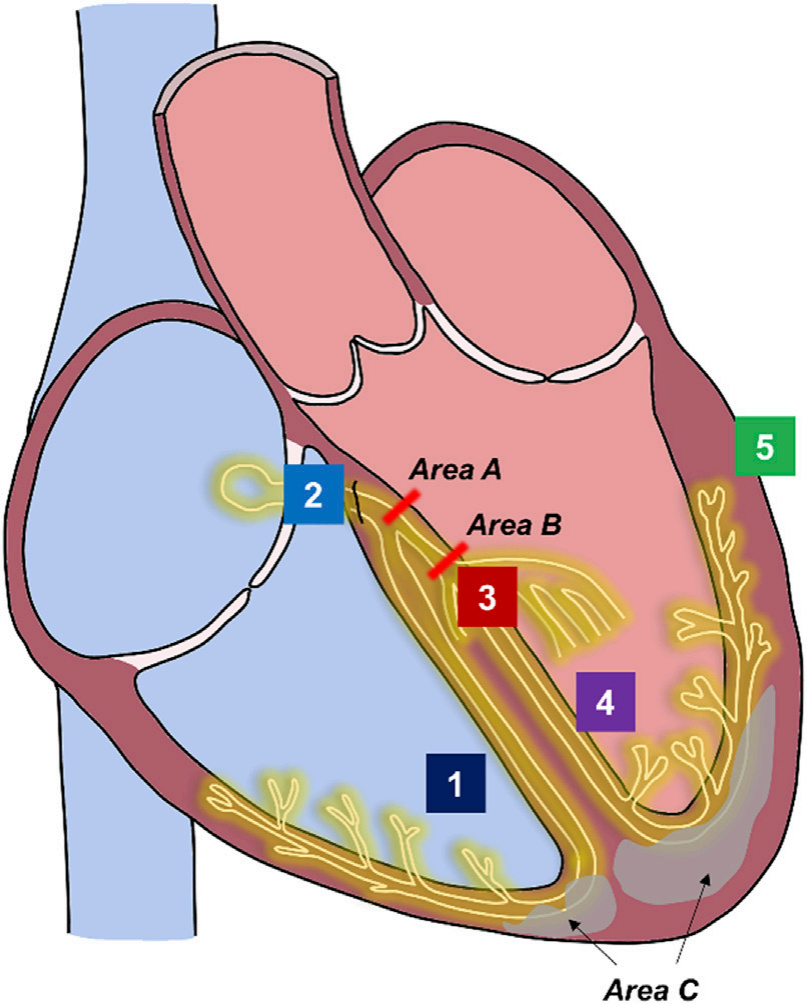
Sites of conduction block in left bundle block pattern and sites of pacing for cardiac resynchronization therapy. Underlying pathophysiology of left bundle block pattern. Course of His-Purkinje system is shown in yellow. Fibrosis within the myocardium in illustrated in gray. Complete conduction block (His fibers or proximal left-sided conduction): areas A or B. Interventricular conduction delay from hypertrophy or fibrosis with intact Purkinje activation: area C. Mixed or multi-level block: areas A + area B + area C. Possible pacing sites for cardiac resynchronization therapy. Site 1: Right Ventricular endocardium pacing (lead tip does not reach conduction system). Site 2: His Bundle pacing (lead tip at or adjacent to His fibers). Site 3: Left bundle branch area pacing (lead tip at or adjacent to left-sided conduction system fibers). Site 4: Left septal pacing (lead tip at left ventricular endocardial surface). Site 5: Left ventricular epicardial pacing. Biventricular pacing: Site 1 and Site 5 or fusion with right bundle activation. Conduction system pacing: Site 2 OR Site 3. Site 4 may also engage fast fibers at the left ventricular endocardial surface. His-optimized cardiac resynchronization therapy: Site 2 in combination with Site 5. Left bundle branch optimized cardiac resynchronization therapy: Site 3 in combination with Site 5.

Intracardiac electroanatomic mapping in patients with LBBB patterns on surface ECG have found heterogenous activation sequences of His-Purkinje conduction, ranging from CCB to IVCD with IPA (Figure 1) (Upadhyay et al., 2019a). CCB comprises of proximal block at the level of the left-sided His fibers (“intrahisian”) and slightly more distal block at the level of left-sided Purkinje fibers (“true left bundle branch block”). In patients who have wide QRS durations but intact His-Purkinje activation on intracardiac mapping, it is hypothesized that the wide QRS duration is from diffuse myopathy, mostly from hypertrophy or fibrosis. It is believed that both processes may be present simultaneously as well (“mixed block”).

## Cardiac resynchronization therapy approaches based on LBBB anatomic site

### Biventricular pacing

CRT for patients with heart failure and LBBB is achieved through placement of both a right ventricular endocardial lead and a left ventricular epicardial lead that is located in the coronary sinus (Figure 1). BiVP is the most robustly studied approach to CRT with multiple large, randomized control trials showing consistent clinical and mortality benefit in patients with severe, clinical heart failure and wide QRS durations with LBBB patterns. The Multisite Stimulation in Cardiomyopathies (MUSTIC), Pacing Therapies in Congestive Heart Failure (PATH-HF), and Multicenter InSync Randomized Clinical Evaluation (MIRACLE) trials found that CRT compared to no CRT improved quality of life metrics (Abraham et al., 2002; Auricchio et al., 2002; Linde et al., 2002). The Comparison of Medical Therapy, Pacing and Defibrillation in Heart Failure (COMPANION) trial, the Cardiac Resynchronization-Heart Failure (CARE-HF) study, the Multicenter Automatic Defibrillator Implantation Trial with Cardiac Resynchronization Therapy (MADIT-CRT), and the Resynchronization/Defibrillation for Ambulatory Heart Failure (RAFT) trial found that CRT reduced death and hospitalizations (Bristow et al., 2004; Cleland et al., 2005; Cleland et al., 2006; Moss et al., 2009; Tang et al., 2010).

Taken together, these landmark clinical trials have firmly established CRT via BiVP as the standard of care for patients with poor systolic function and a wide QRS duration on surface ECG. This is reflected in multi-societal guidelines which give a class I indication for CRT in this subset of patients (Kusumoto et al., 2019; Glikson et al., 2021). Currently, the use of BiVP for CRT should be considered in all patients with LBBB pattern on surface ECG, regardless of the underlying anatomic physiology of LBBB given the numerous large, randomized controlled trials showing significant clinical benefit. With that noted, roughly 30% of patients who undergo CRT do not improve after therapy (Auricchio and Prinzen, 2011). The high number of CRT “non-responders” to BiVP led some experts to consider other locations for pacing, such as CSP and hybrid pacing.

### Conduction system pacing

CRT via CSP relies on inserting a LV lead into IVS to capture in the area of the His bundle (“His bundle pacing”, HBP) or to the left bundle of the Purkinje fibers (“left bundle branch area pacing”, LBBAP) (Figure 1). CSP attempts to counteract cardiac dyssynchrony by capturing sites distal to proximal block to re-engage the native His-Purkinje system and thus shorten QRS. The feasibility of HBP in lieu of an LV lead was shown in 2017 by Ajijola and collegues who successfully completed His-CRT in 16 of the 21 (76%) enrolled patients (Ajijola et al., 2017). Significant improvements in LV ejection fraction (LVEF) and functional class were noted. The His-SYNC pilot study was the first prospective, multi-center, single-blinded randomized, controlled trial to evaluate HBP compared to BiVP for CRT. Among patients with HF who met class I or II guideline indications for CRT, both HBP and BiVP showed improvement in LVEF at 6 months but were not significantly different between the two groups. HBP did significantly reduce QRS duration when compared to BiVP. Importantly, the trial revealed high rates of cross-over as a limitation to HBP as a means to deliver CRT, and that patients with IVCD on surface ECG could not be corrected with HBP (Upadhyay et al., 2019b; Upadhyay et al., 2019c). In the largest trial to date, the His-Alternative Study randomized patients with NYHA class II-IV heart failure symptoms, LVEF ≤ 35%, and LBBB pattern by Strauss’ criteria to HBP versus BiVP (Vinther et al., 2021), and reported results which were in line with the His-SYNC pilot. Similarly, intention-to-treat showed no difference between HBP and BiVP, but per-protocol analysis demonstrated that HBP treated patients had significantly higher LVEF compared to BiVP at 6 months. Rates of procedural success were higher as patients with IVCD were excluded. Improvement in clinical and physical outcomes were similar between groups.

More recently, left bundle branch area pacing (LBBAP) was reported as a novel means to pursue CSP, with reduced procedural complexity and more stable capture thresholds than HBP (Huang et al., 2017). LBBAP as an alternative for HBP was shown by Huang and colleagues in 2020 who successfully implemented LBBAP in 97% of patients with non-ischemic cardiomyopathy, complete LBBB, LVEF ≤ 50% with a CRT indication or who failed to achieve acceptable HBP thresholds (Huang et al., 2020). There was a significant improvement in LVEF and NYHA functional class and 75% achieved normal LVEF (≥ 50%) at 1 year follow-up. The LBBAP Collaborative Study Group retrospectively analyzed over 300 patients who underwent LBBAP and found significant improvement in LVEF with 72% of patients having a clinical response and 73% of patients having an echocardiographic response (Vijayaraman et al., 2021). Finally, LBBAP was compared to HBP and BiVP in a single center, prospective observation study of 127 patients with LVEF ≤ 40%, LBBB, and a CRT indication (Wu et al., 2021). LBBAP improved LVEF and NYHA functional class at a significantly higher rate than BiVP but inferences are limited given the lack of randomization.

While the results of CSP appear promising, there remains a lack of high quality, large, randomized, control trials with blinded assessment of key endpoints and an emphasis on clinical outcomes. Notably, the majority of data reported on CSP have explored echocardiographic endpoints. Given the anatomic consideration of LBBB, it is also worth noting that CSP is likely not appropriate for patients with IPA. Upadhyay et al. found that no patients with IPA had correction of wide QRS duration with HBP (Upadhyay et al., 2019a). This has led some experts to advocate that intracardiac mapping should be employed for patients undergoing CSP. If intracardiac mapping reveals that the patient has IVCD with IPA, then it is argued that CSP should not be used. This would ultimately help to refine patient selection of CSP with the potential to reduce the high cross-over numbers seen in previous RCTs (Tung and Upadhyay, 2020). It is worth noting that BiVP overcomes the anatomic considerations of the LBBB and IPA as it paces directly on the LV epicardium. While there are limited early data suggesting that CSP may have a role in treating nonresponse to BiV nonresponse to CSP is a salient concern which remains to be characterized (Vijayaraman et al., 2022).

### Hybrid pacing

Hybrid methods of pacing have been developed which use sequential or simultaneous fusion of CSP pacing with traditional coronary sinus leads. His-optimized cardiac resynchronization therapy (HOT-CRT) or left bundle branch optimized cardiac resynchronization therapy (LOT-CRT) use a LV epicardial lead with the addition of a His-bundle lead or a left bundle area lead, respectively, to achieve the shortest QRS width. The first experience of HOT-CRT was reported in 2019 by the HIS-Sync Investigators in patients whom successful QRS correction could not be achieved with HBP alone (Upadhyay et al., 2019c). HOT-CRT was successfully implemented in 25 of 27 patients and clinical response was noted in 84% of patients and an echocardiographic response in 92% of patients. Zweeink et al. found that HOT-CRT decreased LV activation time to a significantly greater extent than HBP or BiV pacing alone (Zweerink et al., 2021). LOT-CRT was first reported on by Jastrzębski et al., in 2021 who found that there was improvement in LVEF and reduction in NYHA function class at 3-months follow-up (Jastrzębski et al., 2022). It is also worth noting that in the study, LOT-CRT reduced QRS duration to a greater extent than BiVP or LBBAP. Hybrid methods of pacing appear to show early promise, particularly for patients with mixed block or conduction block on multiple levels.

## Conclusion

Intracardiac EP studies of patients with wide QRS duration and LBBB pattern on surface ECG have demonstrated three distinct entities: CCB, IVCD with IPA, or mixed blocks. The heterogeneity of the underlying pathophysiology of LBBB pattern on surface ECG has pushed investigators to study other forms of assessing LV dyssynchrony or CRT response such as intracardiac recordings, TTE, UHF-ECG, SPECT, and CMR. The highest quality data support CRT via BiVP for patients with clinical heart failure and wide QRS durations. Alternative pacing strategies such as CSP, HOT-CRT, and LOT-CRT show early clinical promise but have yet to show improvement in hard clinical outcomes compared to BiVP. CSP should be reserved for patients with CCB while hybrid models of pacing may be suitable for patients with IPA or mixed levels of conduction block.
